# Clinical and radiological implications of subpotent generic fingolimod in multiple sclerosis: a case series

**DOI:** 10.1177/17562864241300047

**Published:** 2024-11-21

**Authors:** Darin T. Okuda, Lauren M. Tardo, Crystal M. Wright, Shanan B. Munoz, Tom G. Punnen, Mahi A. Patel, Tatum M. Moog, Katy W. Burgess

**Affiliations:** Department of Neurology, Neuroinnovation Program, Multiple Sclerosis & Neuroimmunology Imaging Program, The University of Texas Southwestern Medical Center, 5323 Harry Hines Boulevard, Peter O’Donnell Jr. Brain Institute, Dallas, TX 75390, USA; The University of Texas Southwestern Medical Center, Department of Neurology, Neuroinnovation Program, Multiple Sclerosis & Neuroimmunology Imaging Program, Dallas, TX, USA; The University of Texas Southwestern Medical Center, Peter O’Donnell Jr. Brain Institute, Dallas, TX, USA; The University of Texas Southwestern Medical Center, Department of Neurology, Neuroinnovation Program, Multiple Sclerosis & Neuroimmunology Imaging Program, Dallas, TX, USA; The University of Texas Southwestern Medical Center, Peter O’Donnell Jr. Brain Institute, Dallas, TX, USA; The University of Texas Southwestern Medical Center, Department of Neurology, Neuroinnovation Program, Multiple Sclerosis & Neuroimmunology Imaging Program, Dallas, TX, USA; The University of Texas Southwestern Medical Center, Peter O’Donnell Jr. Brain Institute, Dallas, TX, USA; The University of Texas Southwestern Medical Center, Department of Neurology, Neuroinnovation Program, Multiple Sclerosis & Neuroimmunology Imaging Program, Dallas, TX, USA; The University of Texas Southwestern Medical Center, Peter O’Donnell Jr. Brain Institute, Dallas, TX, USA; The University of Texas Southwestern Medical Center, Department of Neurology, Neuroinnovation Program, Multiple Sclerosis & Neuroimmunology Imaging Program, Dallas, TX, USA; The University of Texas Southwestern Medical Center, Peter O’Donnell Jr. Brain Institute, Dallas, TX, USA; The University of Texas Southwestern Medical Center, Department of Neurology, Neuroinnovation Program, Multiple Sclerosis & Neuroimmunology Imaging Program, Dallas, TX, USA; The University of Texas Southwestern Medical Center, Peter O’Donnell Jr. Brain Institute, Dallas, TX, USA; The University of Texas Southwestern Medical Center, Department of Neurology, Neuroinnovation Program, Multiple Sclerosis & Neuroimmunology Imaging Program, Dallas, TX, USA; The University of Texas Southwestern Medical Center, Peter O’Donnell Jr. Brain Institute, Dallas, TX, USA

**Keywords:** case series, disease modifying therapy, fingolimod, insufficient active ingredient, multiple sclerosis

## Abstract

An expansion in the availability of generic specialty disease modifying therapies (DMTs) for treatment of multiple sclerosis (MS) has increased recently. Generic specialty medications aim to provide greater access to molecules that alter the disease trajectory at lower costs. The US Food and Drug Administration requires generic products to contain between 90% and 110% of the stated active ingredient and an 80%–125% bioequivalence range. We present the clinical experiences and absolute lymphocyte counts (ALC) trends of six people with MS originally treated with Gilenya^®^ (fingolimod) 0.5 mg who were required to transition to generic fingolimod 0.5 mg by third-party administrators, and the medication content from recovered products. Six individuals with acute clinical exacerbations or disease advancement on MRI were identified during routine scheduled visits from a tertiary care center and consecutively included from January 2024 to August 2024. ALC trends were constructed for each individual during Gilenya^®^ and generic fingolimod treatment. These individuals experienced signs of disease advancement while on generic fingolimod 0.5 mg at approximately 1 year of treatment and elevations in ALC, a biological metric related to the mechanism of action of sphingsine-1-phosphate receptor modulation, were observed following the transition. High purity fingolimod for standardization tests, Gilenya^®^ 0.5 mg, and five recovered generic fingolimod 0.5 mg products were independently tested in an accredited laboratory. Gilenya^®^ 0.5 mg capsules had an average fingolimod content of 97.7% (standard deviation (SD) = 2.59%). Three recovered generic fingolimod 0.5 mg products used during relapses had an average content of 91.2% (3.25%), 81.6% (6.24%), and 72.5% (2.05%). Two generic fingolimod 0.5 mg products not associated with relapse activity revealed averages of 97.4% (1.82%) and 103.3% (3.77%). Subpotent generic specialty DMTs may not only result in greater risk for disease activity but may also expose individuals to the potential for disease rebound, depending on the mechanism of action.

## Introduction

Multiple sclerosis (MS) is a chronic, autoimmune condition that falls within the spectrum of central nervous system (CNS) demyelination, affecting nearly 1 million individuals in the United States.^1,2^ The use of disease modifying therapy (DMT) treatment taken orally or parenterally in the management of those diagnosed is fundamental to reducing the risk for permanent neurological disability. Such treatments aim to suppress acute clinical relapses, the development of contrast enhancing and new and/or newly enlarging T2-weighted hyperintense lesions on MRI that may be symptomatic or asymptomatic when present, and the rate of disability accumulation.

At present, there are nine major classes of DMT by mechanism of action (interferon, copolymer, alpha-4 integrin inhibition, sphingosine-1-phosphate receptor modulation, dihydroorotate dehydrogenase inhibition, fumaric acid, anti-CD52, purine antimetabolites, and anti-CD20), yielding nearly 20 brand name agents. Over the past few years, an expanded use in the amount of generic glatiramer acetate, fingolimod, dimethyl fumarate, and teriflunomide has been observed globally, primarily due to patent dispute or expiration. Differing regulatory standards across countries impact generic drug development, from flexible enforcement of quality control measures in certain regions to stringent manufacturing practice requirements set by the European Medicines Agency. Within the United States (US), these treatments are required to meet two standard benchmarks. First, the US Food and Drug Administration (FDA) requires generic products to contain between 90% and 110% of the stated active ingredient. This parameter is set to provide some degree of consistency in the therapeutic effect along with an appropriate level of safety. Second, an 80%–125% bioequivalence range, the area under the plasma concentration-time curve and peak plasma concentration range, is required. Intuitively, if a generic product falls short in meeting either of these standards, there would be risk of inadequate treatment effect and subsequent risk of disease worsening.

The pivotal trials for fingolimod (Gilenya^®^) primarily evaluated the benefits of a once daily oral sphingosine-1-phosphate receptor modulator in reducing the annualized relapse rate in people with relapsing MS.^3,4^ The molecule was also found to be effective in suppressing MRI advancement and clinical disability when compared to placebo. Gilenya^®^ 0.5 mg by mouth daily was approved for use in those with MS on September 22, 2010, and an expanded label was granted on May 11, 2018, for use in children and adolescents age 10 years and older following pivotal data.^5^ The US FDA approved generic fingolimod for the treatment of relapsing forms of MS on December 5, 2019. Due to the availability of a generic alternative, third-party administrators have switched people with MS from Gilenya^®^ over to generic fingolimod, despite disease stability. Recent data suggested that the use of generic fingolimod may not be as effective in suppressing disease advancement and that a higher frequency of side effects may be present in comparison to treatment with Gilenya^®^.^6^ Furthermore, suboptimal content was identified in two of four generic fingolimod products tested, with one having 76.8% and the other 85.5% of the labeled claim.^7,8^ Although out-of-specification findings were identified in the samples tested, the impact of substandard generic fingolimod on clinical measures of disease remain unknown. Recently, underdosed teriflunomide, having an average content of 55% of the labeled active ingredient, was found to be associated with MS disease advancement in comparison to when the individual was stable on Aubagio^®^.^9^

In this report, we describe the clinical experience of a series of individuals with suboptimal outcomes in association with a transition to generic fingolimod along with supporting para-clinical data. We also provide data related to the content of fingolimod relative to the stated label in recovered medications used by individuals with MS who experienced unexpected breakthrough disease. Strategies on how to effectively educate both healthcare providers and people with MS are also provided given the anticipated increase in the use of generic specialty medications within the field.

## Methods

The study group was obtained from existing people with MS receiving care at The University of Texas Southwestern (UTSW) Medical Center in Dallas, TX, USA. Individuals reported here were identified during routine scheduled visits and consecutively included from January 2024 to August 2024, comprising those on generic fingolimod who experienced recent acute exacerbations and/or disease advancement on MRI. The study was performed in alignment with the Strengthening of the Reporting of Observational Studies in Epidemiology guidelines (see online Supplemental Material).

High-purity fingolimod for standardization tests (Selleck Chemicals, Houston, TX, USA), Gilenya^®^ (0.5 mg), generic fingolimod (0.5 mg) acquired from three individuals (Person B (FR1), C (FR2), and D (FR3)), and two generic fingolimod (0.5 mg) products (Generic F1 and F2) not associated with disease advancement that were acquired from an existing specialty pharmacy recovery program^10^ were submitted for testing at an FDA registered, Drug Enforcement Agency registered and International Organization for Standardization 17025:2017 accredited laboratory. Each fingolimod product studied originated from a unique manufacturer. All medications tested were in-date, or not expired, and sealed by the manufacturer prior to sampling, apart from the unsealed medication recovered from our patients. A total of 10 capsules were selected from each bottle and submitted to the independent laboratory for high-performance liquid chromatography testing.

## Results

**Figure 1.**
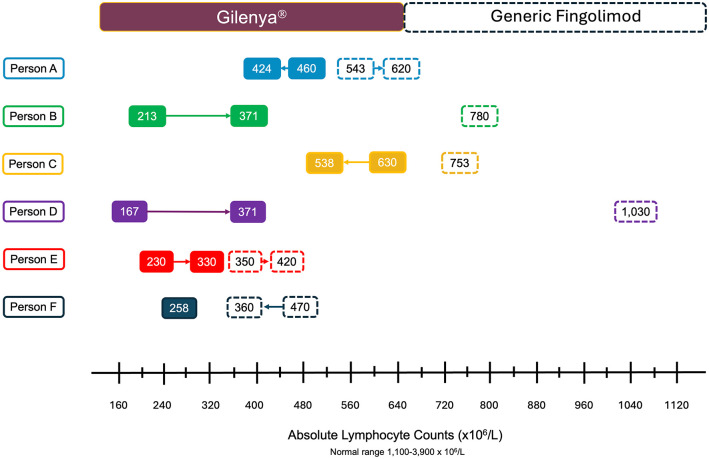
ALC values and trends during treatment with Gilenya^®^ (solid rounded boxes) and generic fingolimod (dashed rounded boxes) by individual experience. The arrows between each box depict the trends over time of ALC for each individual person while on Gilenya^®^ and generic fingolimod. ALC presented for generic fingolimod represents available data near the time of relapses. ALC, Absolute lymphocyte count

**Figure 2.**
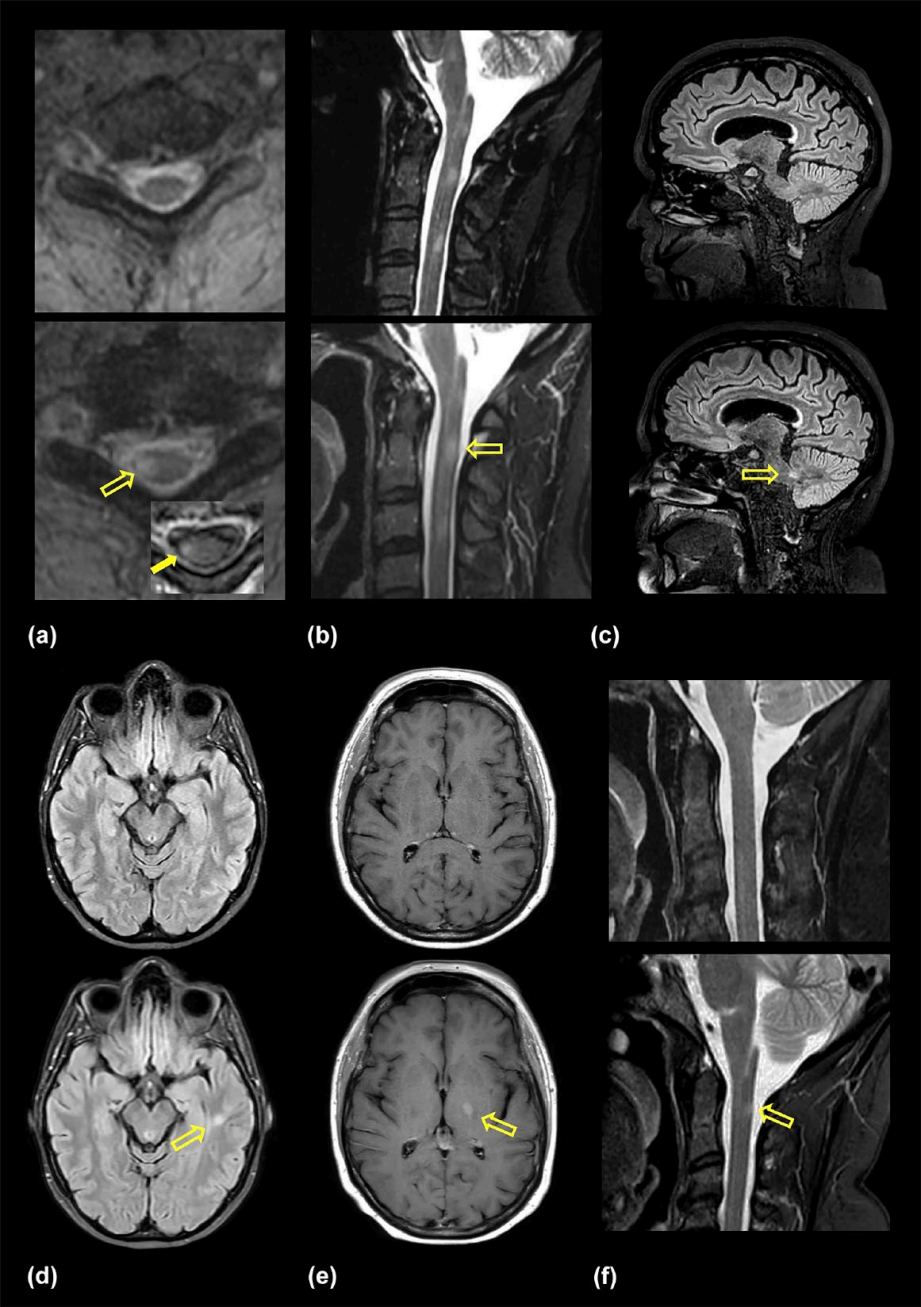
Magnetic resonance imaging data from the six people with MS included in this report with A-F providing MRI evidence of disease advancement. (a) Axial T2 MRI of the cervical spinal cord before (upper panel) and after relapse demonstrating a new T2-weighted hyperintense lesion (lower panel; open yellow arrow) with gadolinium enhancement (inset, lower panel image (solid yellow arrow)). (b) Sagittal short tau inversion recovery (STIR) sequence of the cervical spinal cord demonstrating enlargement of the prior intramedullary lesion at C2 (lower panel; open yellow arrow). Gadolinium was not administered during this study. Note the increase in the T2-weighted signal and caliber of the spinal cord when comparing the upper and lower panels. (c) Sagittal 3-dimensional FLAIR image revealing the presence of a new cerebellar peduncle T2-weighted focus (lower panel; open yellow arrow) when compared to prior MRI data (upper panel). (d) Axial FLAIR images of the brain revealing a new left temporal white matter lesion (lower panel; open yellow arrow). (e) Postgadolinium T1-weighted images highlighting the presence of a deep gray matter (basal ganglia/internal capsule) contrast enhancing lesion (lower panel; open yellow arrow) when compared to prior data (upper panel). (f) Sagittal STIR sequence of the cervical spine demonstrating a new intramedullary T2-weighted hyperintense focus at C2 (lower panel; open yellow arrow) when compared to the prior study (upper panel). FLAIR, fluid attenuated inversion recovery; MS, multiple sclerosis; STIR, short tau inversion recovery.

A total of six people with relapsing-remitting MS on generic fingolimod 0.5 mg with prior treatment history with Gilenya^®^ (0.5 mg) who experienced a recent clinical exacerbation were identified. The median age of these individuals was 44.2 years (range: 38.3–53.2 years) and all were female and White. The median duration of treatment with Gilenya^®^ was 6.25 years (range: 3.59–11.34 years). All individuals were free of clinical or MRI relapses from 6 months after treatment start to their switch over to generic fingolimod. Five individuals were exposed to products from different generic manufacturers and with treatment, higher absolute lymphocyte count numbers were observed around the time of relapse (Figure 1). New adverse reactions following treatment with generic fingolimod were not observed. Four clinical relapses were identified with three events localizing to the spinal cord and one to long tract motor fibers. Among these symptomatic relapses, one individual developed a new spinal cord lesion, another was observed with enlargement of an existing lesion within the cervical spine, and the remaining two had gadolinium enhancing lesions. There were also two MRI relapses observed in the absence of symptoms. Table 1 provides a summary of the baseline and clinical data of included individuals, and Figure 2 provides the findings from the longitudinal MRI data.

**Table 1.** Baseline demographic and clinic information for individuals experiencing a clinical event following a treatment switch to generic fingolimod.

Person	Age (years)	Sex	Race	Ethnicity	Time from first multiple sclerosis symptom (years)	Gilenya^®^ treatment duration prior to switch to generic fingolimod (years)	Time from generic fingolimod start to relapse (years)	Multiple sclerosis relapse type
A	45.4	Female	White	Non-Hispanic	18.07	3.59	0.91	Spinal cord
B	38.3	Female	White	Non-Hispanic	15.94	11.34	0.35	Spinal cord
C	40.3	Female	White	Non-Hispanic	12.32	3.88	0.89	MRI relapse
D	53.2	Female	White	Non-Hispanic	24.64	11.29	0.62	Long tract motor
E	42.5	Female	White	Non-Hispanic	12.11	6.56	0.96	MRI relapse
F	45.7	Female	White	Non-Hispanic	22.35	0.84	1.17	Spinal cord

Gilenya^®^ 0.5 mg capsules had an average fingolimod content of 97.7% (standard deviation (SD) = 2.59%). Generic F1 (0.5 mg) and F2 (0.5 mg) samples revealed averages of 97.4% (1.82%) and 103.3% (3.77%), respectively. The content associated with the generic fingolimod (0.5 mg) recovered from three individuals with relapses was 91.2% (3.25%), just above the 90% threshold; however, four of ten randomly selected capsules were found to be subpotent (89.5%, 89.4%, 87.4%, and 87.0%) (Figure 1 (Person B), Figure 2(b); sample FR1). The second and third recovered generic products revealed a content of 81.6% (6.24%) (Figure 1 (Person C), Figure 2(c); sample FR2) and 72.5% (2.05%) (Figure 1 (Person D), Figure 2(d); sample FR3), respectively. Table 2 summarizes the results obtained from products tested.

**Table 2.** Summary of test results from fingolimod agents studied.

Product	Subject number	Manufacturer	Percent of labeled content (standard deviation)^a^
Gilenya^®^ (0.5 mg)	–	Novartis AG	97.7 (2.59)
Nonrelapse associated			
Generic fingolimod (0.5 mg)			
Generic fingolimod 1 (F1)	–	Generic Manufacturer 1	97.4 (1.82)
Generic fingolimod 2 (F2)	–	Generic Manufacturer 2	103.3 (3.77)
Relapse associated			
Generic fingolimod (0.5 mg)			
Generic fingolimod 1 (FR1)	Person B	Generic Manufacturer 3	91.2 (3.25)
Generic fingolimod 2 (FR2)	Person C	Generic Manufacturer 4	81.6 (6.24)
Generic fingolimod 3 (FR3)	Person D	Generic Manufacturer 5	72.5 (2.05)

a Percent content of stated active ingredient, fingolimod 0.5 mg.

## Discussion

In this report of consecutive people identified who were switched by third-party administrators from Gilenya^®^ to generic fingolimod, we detected the occurrence of clinical and MRI exacerbations along with clear differences in absolute lymphocyte count values. We detected a deficiency in the average content based on US FDA requirements for the generic fingolimod (0.5 mg) recovered from two individuals in which the medication was taken during the time of their exacerbation. In a third recovered sample, 4 of the 10 randomly selected capsules tested were identified as being subpotent with the average content of all samples marginally surpassing the minimal standard. Gilenya^®^ and two other generic fingolimod products, recovered from an existing study in our center, were identified with content within the 90%–110% requirement.

The clinical course of MS is highly variable as individuals may lack the classic symptoms of MS even in the presence of brain or spinal cord anomalies suggestive of inflammatory demyelination.^11–14^ In general, the observation of new T2-weighted hyperintense lesions or gadolinium enhancement in the absence of clinical symptoms is substantially more common than acute clinical events.^15^ Established factors that may increase risk for a clinical exacerbation include the postpartum period^16,17^ along with treatment interruption related to pregnancy or other factors.^18–20^ Intuitively, exposure to subpotent DMTs may also result in an elevated risk for disease advancement. Recognizing alterations in biomarkers related to the treatment effect of a given DMT based on the primary mechanism of action may provide additional evidence of inadequate treatment.

The primary mechanism of action of fingolimod involves preventing the egress of lymphocytes from lymph nodes. This is accomplished following phosphorylation of fingolimod and subsequent interaction with four of five known sphingosine-1-phosphate receptors.^21^ As a consequence, fewer potential autoreactive lymphocytes would have the opportunity to traverse into the brain and spinal cord space, thereby reducing the risk of autoimmune inflammatory injury. This treatment effect was clearly seen in the early study of Gilenya^®^ versus intramuscular interferon beta-1 alpha with mean lymphocyte counts of 0.5 (standard deviation: 0.31) 10^9^/L observed at month 12.^22^ All six individuals described within this report were exposed to generic fingolimod for approximately 1 year and clear differences in the ALC were observed when transitions occurred from Gilenya^®^, suggesting clear disparities in the biological effect of the generic products from five unique generic manufacturers. Ensuring that generic agents exert a meaningful biological effect is critical as with sphingosine-1-phosphate modulators, there is a clear risk of disease rebound.^23^ Those at greater risk were younger in age having a history of greater disease activity prior to cessation, were on treatment for a longer period of time, were identified with lower lymphocyte count values or were switched to a lower efficacy treatment.^24–26^ The observation of relapses within a similar period of time among all individuals described in this report suggests that exposure to subpotent fingolimod places individuals at additional harm due to the variable release of immune cells from lymphatic tissue, resulting from the primary mechanism of action of the molecule.

The FREEDOMS trial evaluated the efficacy of two doses of fingolimod (0.5 mg and 1.25 mg) versus placebo over 24 months.^3^ Both doses were found to be superior to placebo in reducing annualized relapse rates, disability progression, and MRI measures of disease. However, an amendment to the study protocol discontinuing the use of the 1.25 mg dose later followed due to adverse event concerns and lack of substantial benefit in efficacy when compared to the 0.5 mg dose.^27^ But what is the minimally effective dose of fingolimod in adults? In a prior study of individuals between the ages of 18–65 years, the reduction in annualized relapse rate was studied between fingolimod 0.5 mg, glatiramer acetate 20 mg, and fingolimod 0.25 mg.^28^ Fingolimod 0.25 mg demonstrated a numerical reduction in the aggregate annualized relapse rate (14.6%) compared to glatiramer acetate, findings that failed to reach statistical significance. Generic fingolimod products taken by individuals with relapses in our study demonstrated an absolute reduction of 6.5%, 16.1%, and 25.2% when compared to the content of Gilenya^®^ studied in this report. Our findings suggest that doses lower than 0.5 mg may result in reduced clinical efficacy, as seen with fingolimod 0.5 mg versus 0.25 mg, but not with 1.25 mg versus 0.5 mg.^3^

Recent data suggest that lack of effective control of disease results in 50% of DMT treatment switches.^10^ However, treatment switches are more commonly due to nonmedical (i.e., high out-of-pocket costs, desire to minimize daily medication use, preference to switch to a newly released medication, etc.), or tolerability factors (i.e., injection site reactions, hair thinning, gastrointestinal side effects, etc.). These findings may, in part, reflect the influence of treatment algorithms rather than the individualized recommendations provided by clinicians.

The lack of certainty surrounding the integrity of a generic DMT used by people with MS creates clinical challenges. If disease breakthrough occurs with a product containing a substandard amount of active ingredient, the conclusion drawn by healthcare providers would be inadequate control of disease. This may prompt an escalation to a higher efficacy DMT, exposing the person with MS to an increased risk of adverse reactions both during the time of use of the new treatment and even after discontinuation. Switching treatments may also result in higher healthcare costs to both the person receiving treatment and the healthcare system, due to expenses associated with infused therapies and the required surveillance testing.

The retail price between Gilenya^®^ and available generics is not substantially different (Table 3). This prompts the question: Why would generic agents be favored over the branded product? The answer can be found within the profit margins. In August 2024, Gilenya^®^ had an annual retail price of $153,000. Novartis, the manufacturer of Gilenya^®^, may offer an undisclosed rebate, reducing this annual price with the magnitude of the discount remaining confidential. If a 30% rebate is offered, the annual price to a pharmacy benefit manager would be reduced to $107,100. For individuals with commercial insurance, the profit margin is derived from the difference between the cost to acquire the medication and the price charged to employers. If the cost of acquiring the generic version falls below $1000 for annual treatment, third-party administrators and pharmacy benefit managers stand to gain significantly higher profits, having a margin of $133,185.36 (calculated from the average generic cost of $134,185.36 minus $1,000 (generic acquisition cost)) in comparison to the margin of $45,900 driven by rebates.

**Table 3.** Annual and average wholesale prices of U.S. Food and Drug Administration approved Gilenya^®^ and generic manufacturers of fingolimod.

Drug	Manufacturer	Annual average wholesale price
Gilenya^®^ (0.5 mg)	Novartis AG	$155,219.17^a^
Generic fingolimod (0.5 mg)	Accord Healthcare Inc.	$135,109.13^a^
	Apotex Corp.	$135,109.13^a^
	Ascend Laboratories	$127,938.34^a^
	Aurobindo Pharma USA, Inc.	$127,938.34^a^
	AvKARE Inc.	$135,109.13^a^
	Camber Pharmaceuticals Inc.	$127,983.97^a^
	Dr. Reddy’s Laboratories Inc.	$135,109.13^a^
	Glenmark Pharmaceuticals Inc., USA	$135,109.13^a^
	Mylan N.V.	$135,109.13^b^
	Quallent Pharmaceuticals	$135,109.13^a^
	Rising Pharmaceuticals	$135,109.13^a^
	Teva Pharmaceuticals Inc.	$144,566.89^a^
	Zydus Pharmaceuticals Inc.	$135,109.13^a^

a Average wholesale prices acquired from Micromedex^®^ (electronic version). Merative, Ann Arbor, Michigan, USA. RedBook online. Available at: https://www.micromedexsolutions.com/ (cited: August 8, 2024).

b Average wholesale price acquired from Cardinal Health^®^ (electronic version). Cardinal Health Inc. Dublin, OH, USA. Order Express. Available at: https://orderexpress.cardinalhealth.com/ (cited: August 8, 2024).

The emergence of direct-to-consumer online cost-saving solutions has enabled the general public to acquire generic DMTs more conveniently and, at times, at a lower cost as compared to accessing third-party administrator benefits. However, a lack of consistent inventory is a recurring theme for those with MS that we care for in our clinic. Local pharmacies may even resort to acquiring smaller batches of medications from different manufacturers through reverse distribution, introducing variability in the quality of the products that are dispensed. Recently, we identified a 30-day fill of fingolimod in our region that was comprised of medications from 3 different generic manufacturers with 6 capsules provided by 1 manufacturer, 10 from another, and the remaining balance coming from a third manufacturer.

It is important to highlight that medication content is only one of two components required by the US FDA. An understanding of bioequivalence data for generic DMTs that are currently being used by people with MS who have experienced disease advancement or new/worsening adverse reactions, is unknown. The measure may be significantly impacted by the design of the pill or capsule as well as the internal components. Complex drug delivery systems do exist with a variety of treatments, including one well-known DMT, dimethyl fumarate (Tecfidera^®^).^29–31^ Within our center, the observed experience of greater gastrointestinal adverse reactions and flushing with the use of generic dimethyl fumarate in comparison to Tecfidera^®^ may indicate meaningful differences in how the molecule is released and at what pace within the body. The bioequivalence data from the generic products used by the individuals reported here are unknown but may have also contributed to the clinical data described. While the US FDA mandates that generic drugs demonstrate bioequivalence within specified parameters, the detailed results from these studies are not always fully disclosed to the public, limiting critical review and verification.

Counseling on the potential risk of receiving a substandard generic DMT is becoming increasingly relevant in the day-to-day management of people with MS. Providing information for where to find the name of the manufacturer of the generic DMT on the prescription bottle is important along with keeping track of any changes that may occur from month-to-month. Evaluating the appearance of the tablets or capsules and being attentive to any peculiar odors or even taste is critical for recognizing potential batch or manufacturer deficiencies that may impact safety and efficacy. Moreover, if new, reduced, or worsening adverse reactions are experienced, individuals should be in contact with their healthcare team.

The number of individuals reported here is modest. However, incontrovertible evidence of a deficiency in fingolimod content was observed in those with relapses. In individuals where their medication could not be recovered, evidence of elevations in ALC were observed that mirrored those with underdosed fingolimod, suggesting that they may have been exposed to nonbioequivalent products. The testing of recovered generic fingolimod product from all individuals presented here would be ideal; however, many were exposed to more than one generic manufacturer during the year of treatment. In addition, a more comprehensive understanding of the time course of change in absolute lymphocyte count levels would have been better achieved using defined assessment intervals following exposure to generic fingolimod agents in tandem with prospective efforts centered around compliance. The total number of individuals exposed to generic fingolimod within our center is unknown and future studies aimed at verifying these findings are ongoing.

The long-term management of people with MS is complex with treatment plans uniquely designed for each individual. Specialists involved in directing care are now faced with an added factor in management models, potential deficiencies or inconsistencies in the quality of medications that are being dispensed. In general, value exists with generic DMTs for the management of MS and as shown here, not all generic products were deficient in content. However, being able to assess the quality of products used by consumers before clinical events occur remains a central goal given the risk of relapse with incomplete recovery and permanent neurological deficits associated with subtherapeutic responses to subpotent therapies. Also, generic fingolimod products that tested within the required FDA range for content may not perpetually be sound given variations in quality control and manufacturing processes. As potential for batch-to-batch differences may exist and with new generic manufacturers emerging, an ideal scenario would be rapid testing for drug quality by healthcare systems or another entity before these treatments are released to the public. Additionally, if DMTs are identified to be substandard to US FDA guidelines, the ultimate bearer of the financial costs for the treatments themselves (employers for commercially covered individuals) and the cost for care should not be from people with MS or healthcare systems but from third-party administrators and pharmacy benefit managers responsible for mandating use of these generic specialty medications.

## Supplemental Material

sj-pdf-1-tan-10.1177_17562864241300047 – Supplemental material for Clinical and radiological implications of subpotent generic fingolimod in multiple sclerosis: a case series Supplemental material, sj-pdf-1-tan-10.1177_17562864241300047 for Clinical and radiological implications of subpotent generic fingolimod in multiple sclerosis: a case series by Darin T. Okuda, Lauren M. Tardo, Crystal M. Wright, Shanan B. Munoz, Tom G. Punnen, Mahi A. Patel, Tatum M. Moog and Katy W. Burgess in Therapeutic Advances in Neurological Disorders

## Declarations

**Ethics approval and consent to participate:** This study was approved by the UTSW Institutional Review Board (STU-072016-012) and performed in accordance with the ethical standards laid down in the 1964 Declaration of Helsinki and later amendments. Written informed consent was obtained from all study participants.

**Consent for publication:** Written informed consent was obtained from all study participants.

**Competing interests:** D.T.O. received personal compensation for consulting and advisory services from Biogen Inc., Eisai, EMD Serono, Genentech, Genzyme/Sanofi, Immunic Therapeutics, Moderna, RVL Pharmaceuticals, Inc., and Zenas BioPharma along with research support from EMD Serono/Merck and Novartis. D.T.O. has issued national and international patents along with pending patents related to other developed technologies. D.T.O. received royalties for intellectual property licensed by the Board of Regents of the University of Texas System. D.T.O. is the Founder of Revert Health Inc. L.M.T. received personal compensation for advisory services from EMD Serono. C.M.W. reports personal compensation for advisory services from Cycle Pharmaceuticals, EMD Serono, and Sanofi. S.B.M. reports personal compensation for advisory services from Horizon Therapeutics, Bristol Myers Squibb, Novartis, and TG Therapeutics and research support from Genentech. K.W.B. reports personal compensation for advisory services from Bristol Myers Squibb. T.G.P., M.A.P., and T.M.M. report no disclosures.

**Availability of data and materials:** The datasets generated and analyzed during this study are available from the corresponding author on reasonable request following successful completion of a data sharing agreement with the University of Texas Southwestern Medical Center.
